# Identification of a novel motif responsible for the distinctive transforming activity of human T-cell leukemia virus (HTLV) type 1 Tax1 protein from HTLV-2 Tax2

**DOI:** 10.1186/1742-4690-6-83

**Published:** 2009-09-17

**Authors:** Toshiyuki Shoji, Masaya Higuchi, Rie Kondo, Masahiko Takahashi, Masayasu Oie, Yuetsu Tanaka, Yutaka Aoyagi, Masahiro Fujii

**Affiliations:** 1 Division of Virology, Niigata University Graduate School of Medical and Dental Sciences, 1-757 Asahimachi-Dori, Niigata 951-8510, Japan; 2 Division of Gastroenterology and Hepatology, Niigata University Graduate School of Medical and Dental Sciences, 1-757 Asahimachi-Dori, Niigata 951-8510, Japan; 3 Department of Immunology, Graduate School and Faculty of Medicine, University of the Ryukyus, Uehara 207, Nishihara-cho, Nakagami-gun, Okinawa 903-0215, Japan

## Abstract

**Background:**

Human T-cell leukemia virus type 1 (HTLV-1) is a causative agent of adult T-cell leukemia (ATL), whereas its relative HTLV-2 is not associated with any malignancies including ATL. HTLV-1 Tax1 transformed a T-cell line from interleukin (IL)-2-dependent growth to IL-2-independent growth, with an activity that was much more potent in comparison to HTLV-2 Tax2. This distinction was mediated by at least two Tax1 specific functions, an interaction with host cellular factors through the PDZ domain binding motif (PBM) and the activation of NF-kappaB2 (NF-κB2)/p100.

**Results:**

Using a series of Tax1 chimeric proteins with Tax2, we found that amino acids 225-232 of Tax1, the Tax1(225-232) region, was essential for the activation of NF-κB2 as well as for the high transforming activity. The strict amino acid conservation of Tax1(225-232) among HTLV-1 and simian T-cell leukemia virus type 1 (STLV-1), but not HTLV-2 and STLV-2, indicates that function(s) through the Tax1(225-232) region are biologically significant. Interestingly, another HTLV-1 relative, HTLV-3, has a PBM, but does not conserve the Tax1(225-232) motif in Tax3, thus indicating that these two motifs classify the three HTLVs into the separate groups.

**Conclusion:**

These results suggest that the combinatory functions through Tax1(225-232) and PBM play crucial roles in the distinct biological properties of the three HTLVs, perhaps also including their pathogenesis.

## Background

Human T-cell leukemia virus type 1 (HTLV-1) and HTLV-2 are onco-retroviruses, which immortalize human T-cells *in vitro *and *in vivo *[1,2]. These immortalizations establish life-long persistent infections in the host. However, only the HTLV-1 infection, but not the HTLV-2 infection, leads to adult T-cell leukemia (ATL), characterized by a massive clonal expansion of the T-cells infected with HTLV-1 [1-3]. Since only a fraction of HTLV-1 infected individuals (approximately 5%) suffer ATL after a long latency period (60 years on average), the genetic and/or epigenetic changes in the HTLV-1 infected T-cells as well as the deterioration of the host immunity are thought to be prerequisites for ATL development [1,2]. Therefore, HTLV-2 infection cannot promote some step(s) in these late event(s).

HTLV-1 and HTLV-2 encode the transforming proteins, Tax1 and Tax2, respectively, whose expression plays a central role in the immortalizations of infected T-cells and their persistent infections [2,4-7]. Tax1 has multiple functions in T cells, including the activation of cellular genes through the transcription factors NF-κB, AP-1, SRF, and CREB/ATF, and in the inactivation of several tumor suppressor genes, such as p53 [7-18]. However, these functions do not explain the HTLV-1 specific leukemogenesis, because Tax2 shares them equivalently.

There is one striking difference between Tax1 and Tax2. Tax1 transforms a mouse T-cell line (CTLL-2) from interleukin(IL)-2 dependent growth to independent growth, and the activity was much more potent in comparison to Tax2 [19]. Such activity requires the Tax1-specific activation of the non-canonical NF-κB pathway [20]. NF-κB is a family of transcription factors that share the DNA binding Rel homology domain. It includes p105/p50, p65, c-Rel, p100/p52 and RelB. They are generally classified into two groups, the canonical NF-κB (p105/p50, p65, c-Rel) or the non-canonical NF-κB (p100/p52, RelB) [21]. The canonical NF-κB pathway is typically activated by inflammatory cytokines such as TNFα and IL-1, thus playing roles in inflammation as well as in apoptosis. In comparison, the non-canonical NF-κB pathway is activated by lymphotoxin β, BAFF, and CD40 ligand, thus playing roles in the development and organogenesis of the lymphoid system. Moreover, both pathways are aberrantly activated in various malignancies, including leukemia and lymphoma [22,23].

By using a series of Tax1 chimeric proteins with Tax2, we herein show that the Tax1(225-232) region plays a crucial role in the increased transforming activity seen with Tax1 relative to Tax2, mostly through the activation of the non-canonical NF-κB/p100 pathway. Taking into account the fact that the amino acid sequence of Tax1(225-232) is strictly conserved between HTLV-1 and simian T-cell leukemia virus type 1 (STLV-1) but not with HTLV-2 nor STLV-2, these results suggest that function(s) through Tax1(225-232) play crucial roles in the pathogenicity of HTLV-1.

## Results

### Identification of Tax1 domains responsible for p100 processing

**Figure 1:**
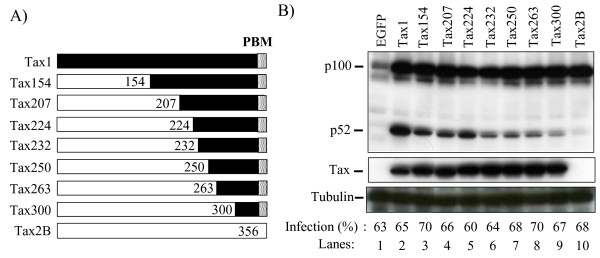
**NF-κB2/p100 processing activities of Tax1 chimeric proteins with Tax2**. (A) The structure of Tax1, Tax2B, and their mutants, and the boundary amino acids of the chimeras are indicated. (B) Jurkat cells were infected with lentiviruses encoding the indicated proteins. The cell lysates were prepared 48 hours after infection, and the amounts of p100, p52, Tax and α-Tubulin in the lysates were measured by Western blotting analysis using anti-p100, anit-Tax1, and anti-α-Tubulin antibodies. EGFP was translated from a bicistronic transcript encoding the *tax *gene, and the infection level (%) was normalized by EGFP expression of the cells infected with the indicated lentiviruses. The anti-Tax1 antibody could not recognize Tax2B protein.

**Figure 2:**
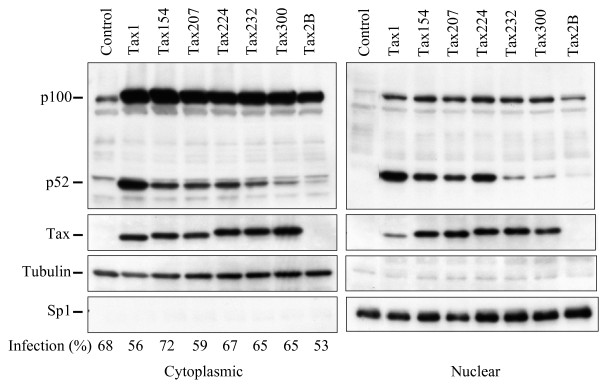
**Tax1 and its mutants induce nuclear localization of p52**. Jurkat cells were infected with lentiviruses encoding the indicated proteins. The cytoplasmic and nuclear lysates were prepared 48 hours after infection, and the amounts of p100, p52, Tax, α-Tubulin, and Sp1 in the cytoplamic and nuclear lysates were measured by Western blotting analysis using anti-p100, anti-Tax1, anti-α-Tubulin, and anti-Sp1 antibodies. EGFP was translated from a bicistronic transcript encoding the *tax *gene, and the infection level (%) was normalized by EGFP expression of the cells infected with the indicated lentiviruses. The anti-Tax1 antibody could not recognize Tax2B protein.

HTLV-1 Tax1, but not HTLV-2 Tax2, through the processing of NF-κB2/p100 into p52, activates the non-canonical NF-κB pathway [20,24]. In order to delineate the domain of Tax1 responsible for NF-κB2/p100 activation, lentiviral vectors expressing a series of Tax1 chimeric proteins with Tax2 subtype B (Tax2B) were used to infect a human T-cell line Jurkat (Fig. 1A). After the normalization of the infections using enhanced green fluorescence protein (EGFP), which was simultaneously expressed from a bicistronic transcript encoding the *tax1 *genes, the amounts of NF-κB2/p100 and its processed product p52 in the infected cell lysates were determined by Western blot analysis using an anti-p100/p52 antibody (Fig. 1B). Tax1 in the Jurkat cells efficiently induced p100 as well as p52 expression relative to the control (EGFP) cells, whereas Tax2 induced only p100 (Fig. 1B, lane 2 and lane 10). It should be noted that the induction of p100 by Tax1 and Tax2 are mediated through the canonical NF-κB pathway as discussed previously, and the activities were equivalent to each other (lane 2 and lane 10) [20]. The chimeric Tax1 proteins showed different p100 processing activities and identified two critical regions of Tax1 which are responsible for p100 processing; the first region is located in the Tax1 amino acids 1-154, Tax1(1-154) (compare lane 2 and lane 3), and the second region is located in the Tax1(225-232) region (compare lane 5 and lane 6). All these chimeric proteins, except for Tax2B, were equivalently detected by an anti-Tax1 antibody in Jurkat cells, and they exhibited an equivalent p100 induction. In addition, anti-Tax2B detected the equivalent expression of Tax2B and Tax300 in Jurkat cells (data not shown) [20]. After processing from p100 into p52, the p52 protein next translocates from the cytoplasm into the nucleus and then either activates or represses transcription [21]. We, thereafter, examined whether Tax1 induces the translocation of p52 into the nucleus by subcellular fractionation assay (Fig. 2). Tax1, but not Tax2B, was thus found to induce the expression of p52 in the nucleus, and the aforementioned two regions of Tax1, Tax1(1-154) and Tax1(225-232), played crucial roles in the translocation of p52.

**Figure 3:**
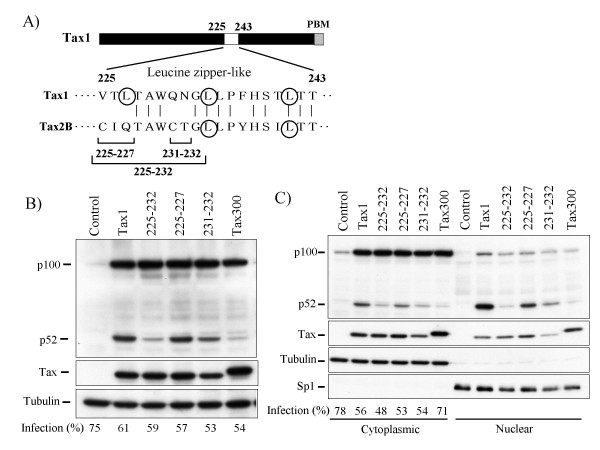
**Tax1(225-232) is involved in the p100 processing**. (A) Amino acid sequence of Tax1(225-243) and Tax2B(225-243). The exchanged amino acids in the respective mutants and leucine residues putatively constituting the leucine zipper (LZ) structure are indicated. (B) Tax1^225-232^, Tax1^225-227^, and Tax1^231-232^ have amino acid substitutions derived from Tax2B in the indicated regions in the backbone of Tax1. Jurkat cells were infected with lentiviruses encoding the indicated Tax or the mutant proteins. The total cell lysate (B), the cytoplasmic and the nuclear lysates (C) were prepared 48 hours after infection, and the amounts of p100, p52, Tax, α-Tubulin, and Sp1 in the lysates were measured by Western blotting analysis using anti-p100, anti-Tax1, anti-α-Tubulin or anti-Sp1 antibodies. The infection was normalized by EGFP expression on FACS analysis, and the infection level (%) was indicated.

Thereafter, we explored the contribution to p100 processing by the Tax1(225-232) region. In this region, Tax1 has five amino acids that are distinct from Tax2B (Fig. 3A). Therefore, they were entirely or partly exchanged with those of Tax2B. The substitution of all five amino acids in Tax1, Tax1^225-232^, prominently reduced the p100 processing activity, and the level was equivalent to that of Tax300 (Fig. 3B). However, the substitutions of only the first three or the last two minimally and partially reduced the activities, respectively, although the amount of Tax1^231-232^ was reproducibly less in comparison to those of Tax1 or Tax1^225-227^. A subcellular fractionation assay showed that the substitution of all five amino acids in the Tax1(225-232) region prominently decreased the nuclear expression of p52 relative to Tax1. The nuclear expression of p52 induced by Tax1^225-227^ and Tax1^231-232^ was also less than that seen with Tax1, but this was more than that observed for Tax1^225-232^ (Fig. 3C). These results suggested that both Tax1(225-227) and Tax1(231-232) are involved in the activation of NF-κB2/p100.

**Figure 4:**
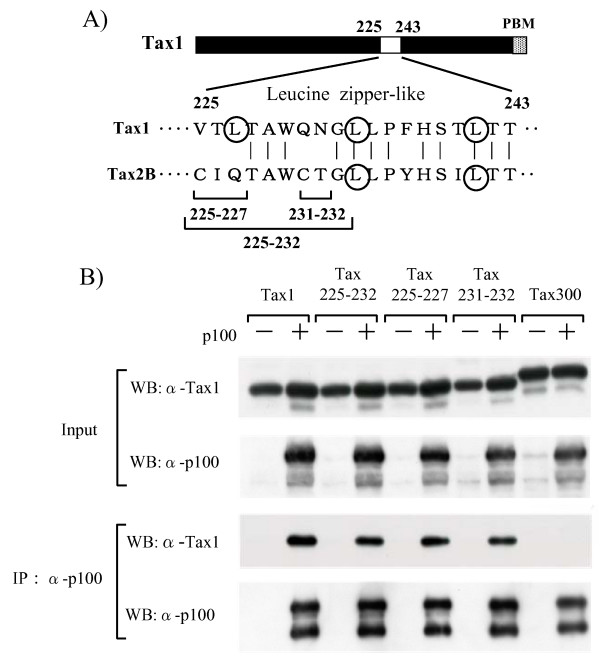
**Tax1 mutants in the Tax1(225-232) region interact with p100**. (A) Amino acid sequence of Tax1(225-243) and Tax2B(225-243). The exchanged amino acids in the respective mutants and leucine residues putatively constituting the leucine zipper (LZ) structure are indicated. (B) 293T cells were transfected with the indicated Tax expression plasmids together with a p100 expression plasmid. At 48 hours following transfection, the cell lysates were prepared and immunoprecipitated with the anti-p100 antibody. The precipitated proteins were characterized by Western blot analysis with anti-Tax1, or anti-p100 antibodies. An aliquot of the lysates, removed before immunoprecipitation, was also characterized as an input (Input).

Tax1, but not Tax2B, is known to interact with p100 and to induce p100 processing [20]. Therefore, Tax1 or the indicated mutant plasmids together with the p100 plasmid were co-transfected into an embryonic kidney cell line 293T. The cell lysates were immunoprecipitated using an anti-p100 antibody, and the immunoprecipitated proteins were characterized with an anti-Tax1 antibody. Consistent with the previous finding, Tax1 but not Tax300 efficiently interacted with p100 (Fig. 4). Similar to Tax1, all three Tax1 mutants in the Tax1(225-232) region were efficiently bound to p100, and the affinities were equivalent to that of Tax1, thus indicating that a function in Tax1(225-232) is required for p100 processing and p52 nuclear translocation which is distinct from the interaction with p100.

#### Tax1(225-232) is required for the increased transforming activity of Tax1 relative to Tax2B

**Figure 5:**
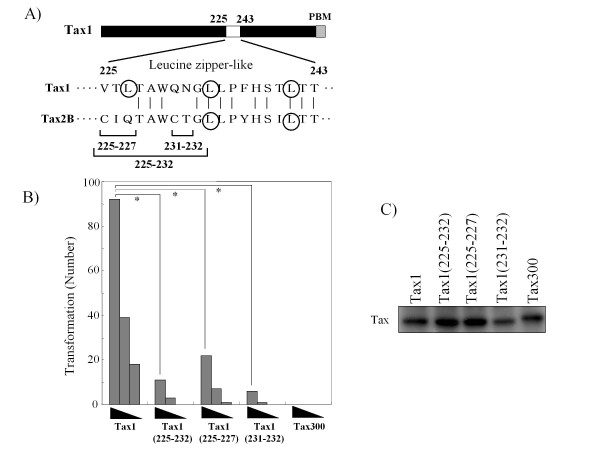
**Tax1(225-232) is required for the increased transforming activity relative to Tax2B**. (A) Amino acid sequences of the 225-234 region of Tax1. (B) CTLL-2 cells were infected with lentiviruses encoding the indicated Tax proteins in the presence of IL-2. At 48 hours after infection, the cells (1 × 10^3^, 3 × 10^3^, and 1 × 10^4^ cells/well) were cultured in 96-well plates without IL-2. After 4 weeks of culture, the wells containing the outgrowing cells were counted using light microscopy. The numbers of positive wells are shown, and the maximum number was 96. The data are representative of three independent experiments. The P values were calculated by chi-square test, and the *P values of Tax1 versus the mutants were < 0.001. (C) The cell lysates prepared at 48 hours after infection were characterized by Western blot analysis probed with the anti-Tax1 antibody.

CTLL-2 is a mouse T-cell line that requires interleukin (IL)-2 for growth. We previously showed that Tax1 transformed CTLL-2 and induced IL-2-independent growth [25], and that the activity was severely diminished by reducing the NF-κB2/p100 protein through RNA interference [20]. In order to examine the role of the Tax1(225-232) region in the transforming activity, CTLL-2 cells were transduced with the lentivirus vectors encoding the Tax1 mutants used above (Fig. 5A), and they were cultured in the absence of IL-2 for four weeks. Tax1, but not Tax300, induced IL-2-independent growth of CTLL-2, consistent with the previous findings (Fig. 5C) [20]. On the other hand, the transforming activities of all three mutants in the Tax1(225-232) region were much lower in comparison to Tax1. The anti-Tax1 antibody showed that Tax1 and the mutants, except for Tax1^231-232^, were equivalently expressed in CTLL-2 cells 48 hours after the infection (Fig. 5B). These results thus suggest that the Tax1(225-232) region plays a crucial role in cellular transformation, most likely through NF-κB2/p100 activation.

### The cryptic NES region of Tax1 negatively regulates the transforming activity

**Figure 6:**
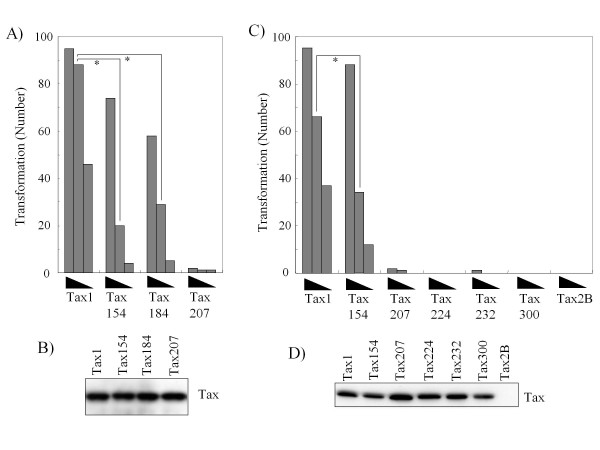
**The transforming activities of the Tax1 chimeric proteins with Tax2B**. (A, C) CTLL-2 cells were infected with lentiviruses encoding the indicated Tax genes in the presence of IL-2. At 48 hours after infection, the cells (1 × 10^3^, 3 × 10^3^, and 1 × 10^4^ cells/well) were cultured in 96-well plates without IL-2. After 4 weeks of culture, the wells containing the outgrowing cells were counted using light microscopy. The number of positive wells is shown; the maximum number was 96. (A, C) The cell lysates prepared at 48 hours after infection (B, D) were characterized by a Western blot analysis probed with the anti-Tax1 antibody. The data are representative of three independent experiments. *The P values were < 0.001.

**Figure 7:**
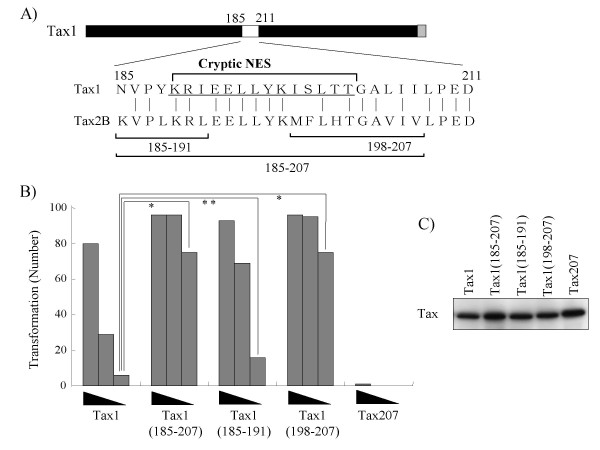
**The Tax1(185-207) region negatively regulates the transforming activity of Tax1**. (A) The amino acid sequences of the 185-207 region of Tax1. The exchanged amino acids in the respective mutants and leucine and isoleucine residues putatively constituting the cryptic NES are indicated. (B) CTLL-2 cells were infected with lentiviruses encoding the indicated Tax genes in the presence of IL-2. At 48 hours after infection, the cells (1 × 10^3^, 3 × 10^3^, and 1 × 10^4^ cells/well) were cultured in 96-well plates without IL-2. After 4 weeks of culture, the wells containing the outgrowing cells were counted using light microscopy. The number of positive wells is shown, and the maximum number was 96. The data are representative of three independent experiments. *The P values and **the P value were < 0.001 and < 0.05, respectively. (C) The cell lysates prepared at 48 hours after infection were characterized by Western blot analysis probed with the anti-Tax1 antibody.

**Figure 8:**
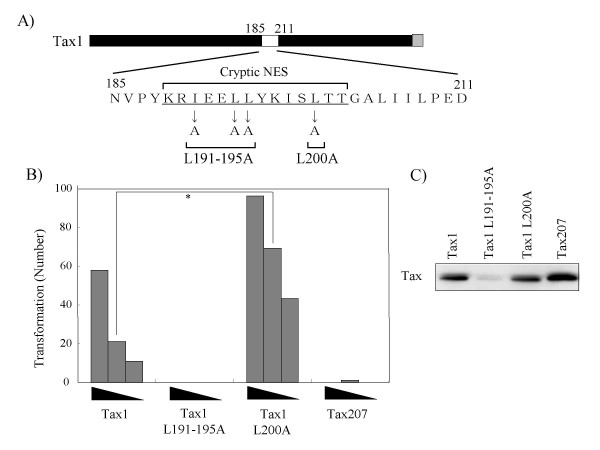
**A Tax1(L^200^-A) mutant exhibits higher transforming activity in comparison to Tax1**. (A) The amino acid sequences of the 185-207 region of Tax1. The mutated amino acids in the respective mutants are indicated. (B) CTLL-2 cells were infected with lentiviruses encoding the indicated Tax genes in the presence of IL-2. At 48 hours after infection, the cells (1 × 10^3^, 3 × 10^3^, and 1 × 10^4^ cells/well) were cultured in 96-well plates without IL-2. After 4 weeks of culture, the wells containing the outgrowing cells were counted using light microscopy. The number of positive wells is shown, and the maximum number was 96. The data are representative of three independent experiments. *The P values were < 0.001. (C) The cell lysates prepared at 48 hours after infection were characterized by a Western blot analysis probed with the anti-Tax1 antibody.

Thereafter, we examined the transforming activities of the Tax1 chimeric proteins characterized in Figure 1. Tax154 and Tax184 showed a much higher transforming activity in comparison to Tax300. However, the activity was reproducibly lower in comparison to Tax1 (Fig. 6). On the other hand, Tax207, with an equivalent p100 processing activity to Tax154 or Tax184, exhibited a deteriorated transforming activity, thus suggesting that Tax1 amino acid 185-207 represents another distinction from Tax2B in the transformation process. To clarify this issue, the amino acids 185-207 in Tax1 were exchanged with those of Tax2B, and the transforming activity was examined (Fig. 7). Unexpectedly, all three Tax1 mutants in this region exhibited transforming activities that were higher in comparison to Tax1. These results suggest that the simultaneous exchange of the Tax1(1-184) and Tax1(185-207) regions with those of the Tax2B regions, but not the exchange of either one, reduces the transforming activity, and that the Tax1(185-207) region by itself has a negative function for the transforming activity. The amino acid sequences of Tax1(185-207) resemble the leucine-rich nuclear export signal (NES). The Tax1 mutants of this motif did not alter the subcellular localization [26]. However, they were found to localize exclusively in the cytoplasm after the deletion of the C-terminal regions to this motif [26]. Based on this information, we changed Tax1 amino acid Leu200 to Ala, which abrogated the cryptic NES function in the previous study (Fig. 8) [26]. Similar to Tax1^185-207^ and Tax1^198-207^, Tax1(Leu^200^-Ala) also exhibited greater transforming activity in comparison to the wild-type protein. Unfortunately, Tax1(L^191-195^-A) was unstable in CTLL-2, and was excluded from consideration. Taken together, these results suggest that the Tax1(185-227) region negatively regulates the transforming activity of Tax1, and the activity might be associated with the cryptic NES function.

## Discussion

**Figure 9:**
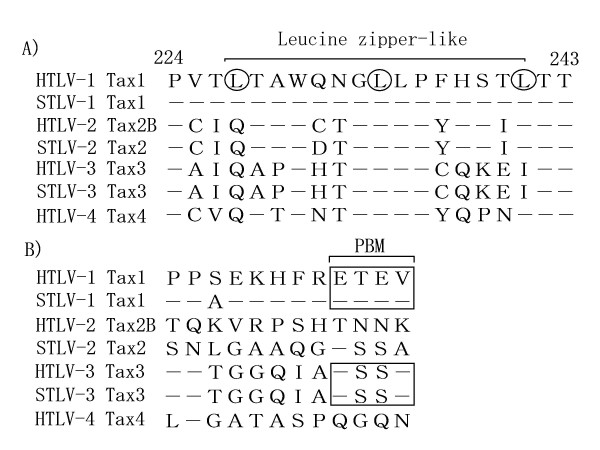
**(A) The amino acid sequences of the Tax(224-243) region from HTLVs and STLVs**. The amino acids of Tax from the other six viruses identical to that of Tax1 are indicated by a bar. The leucine residues constituting a putative LZ structure are surrounded by a circle. The amino acid sequences were obtained from a previous study [35]. (B) The amino acid sequences in the C-terminal ends of the respective Tax protein are shown. The PBMs are surrounded by squares.

We, herein, show that the 225-232 region of Tax1 is crucial for obtaining a greater transforming activity in comparison to Tax2B, measured as IL-2-independent growth of an originally IL-2-dependent cell line CTLL-2, and that this function is mostly mediated through the activation of NF-κB2/p100 (Fig. 3 and 5). Since the amino acid sequence of Tax1(225-232) is strictly conserved in HTLV-1 and STLV-1, but not in HTLV-2 and STLV-2 (Fig. 9A), the present study indicates that the function(s) observed in the Tax1(225-232) region, such as NF-κB2/p100 activation play significant roles in the distinct transforming activity of Tax1 compared to Tax2B, and could thus influence the pathogenesis of HTLV-1.

We initially expected that Tax1(225-232) was involved in the interaction with p100. However, this hypothesis was not supported (Fig. 4). Therefore, it is unclear precisely what role Tax1(225-232) plays in the activation of NF-κB2. We believed that further analyses will provide better insights into the mechanism by which Tax1 activates the alternative NF-κB pathway.

Tax207 exhibited a much reduced transforming activity in CTLL-2 than Tax184 (Fig. 6A), thus suggesting that the Tax1(185-207) region plays a positive role in cellular transformation. However, Tax1^185-207^, a Tax1 mutant with the Tax2B(185-207) region, exhibited slightly higher transforming activity than Tax1 (Fig. 7). Therefore, it is unclear how the Tax1(1-207) region in the context of Tax1 has a positive function for cellular transformation. Alternatively, the Tax2B(1-207) region in the context of Tax207 might possess an inhibitory activity against cellular transformation.

A previous study showed that the Tax1 mutation (L^200^-A) abrogated the cryptic NES activity, which was observed only after the deletion of the C-terminal Tax1 region [26]. The same mutant exhibited a transforming activity higher than wild-type Tax1 (Fig. 8). In addition, three Tax1 chimeric proteins with Tax2B in this NES region also augmented the transforming activity (Fig. 6). Since Tax2 also has the cryptic NES in this region [27], it is unlikely that the NES activity by itself has an inhibitory activity toward the transforming activity. Although the mechanism is unclear in the present study, one feasible explanation is that the cellular factors regulating the Tax1-specific cryptic NES activity has a negative function for the transformation. However, a further analysis is required to establish this mechanism.

Tax2B transforms a rat fibroblast cell line Rat-1, thus causing it to induce colonies in soft agar [28], but this activity was lower in comparison to that of Tax1 [29]. However, unlike CTLL-2, a Tax2B fusion with Tax1 PBM or Tax300 transformed Rat-1 with an equivalent efficiency to Tax1. Therefore, the functions through the Tax1(225-232) region may be constitutively active in Rat-1, or they may not be needed in the transformation of Rat-1. In support of the former hypothesis, NF-κB2/p100 in Rat-1 was found to be constitutively active without Tax1 [30].

Recently, novel HTLV family members HTLV-3 and HTLV-4 were isolated in Africa [31-34], although it is unclear whether HTLV-3 and HTLV-4 are associated with any particular disease such as leukemia. The amino acid sequence and the functional analysis of HTLV-3 Tax3 showed that Tax3 has a functional PBM, and is capable of interacting with a PDZ domain protein [35]. Interestingly, the Tax3(225-232) regions of HTLV-3 as well as simian T-cell leukemia virus type 3 did not show any similarity to that of Tax1, and they were more similar to that of Tax2 (Fig. 9). On the other hand, HTLV-4 Tax4 does not have a PBM, while it also shows a higher amino acid similarity to Tax2 in the Tax(225-241) region than others. Therefore, the PBM and the Tax1(225-241) motif can classify the four HTLVs into three or four separate groups. We believe that the characterizations of these two motifs of Tax will unveil the functions associated with the respective pathogenesis of the different viruses.

## Materials and methods

### Cells and cell culture conditions

CTLL-2 is a mouse cytotoxic T-cell line, the growth of which is dependent on interleukin-2 (IL-2). CTLL-2 cells were cultured in RPMI1640 medium supplemented with 10% fetal bovine serum (FBS), 55 μM 2-mercaptoethanol and 500 pM recombinant human IL-2. Jurkat is a human T-cell line and the Jurkat cells were cultured in RPMI1640 medium supplemented with 10% FBS (RPMI/10%FBS). 293T is a human embryonic kidney cell line and the cells were cultured in Dulbecco's modified Eagle's medium (DMEM) supplemented with 10% FBS.

### Plasmids

The lentiviral Gateway destination vector CS-EF-IG-RfA and the expression vector pEFneo-RfA were previously described [20,36]. The respective tax mutant genes were constructed by the PCR method. They were cloned into pENTR/D-TOPO or pENTR2B (Invitrogen) and transferred into CS-EF-IG-RfA and pEFneo-RfA through an LR recombination reaction using LR clonase (Invitrogen). Tax300 was originally designated as Tax221 in the previous study [28]. The human NF-κB2/p100 expression vectors, pEFneo-p100 was previously described [20,36].

### Lentiviruses

Recombinant lentiviruses were generated by transfecting pCAG-HIVgp, pCMV-VSV-G-RSV-Rev (provided by Dr. H. Miyoshi, RIKEN Tsukuba Institute) and the respective lentiviral vectors encoding Tax1, Tax2B or their mutants into 293T cells using FuGENE 6 (Roche). Forty-eight hours after the transfection, the supernatant was collected and used to infect CTLL-2 or Jurkat cells (4 × 10^5^) in a final volume of 2 ml of RPMI/10%FBS containing 8 μg/ml polybrene with 500 pM IL-2 for CTLL-2 or without it for Jurkat.

### Immunoprecipitation and Western blotting

In order to prepare the total cell extracts, the cells were lysed in sodium dodecyl sulfate (SDS) sample buffer (2% SDS, 62.5 mM Tris-HCl pH 6.8, 10% glycerol, 50 mM dithiothreitol, 0.01% bromophenol blue). For the immunoprecipitation assays, 293T cells were transiently transfected with Tax1 or Tax mutant expression plasmids with or without pEFneo-p100. The cells were lysed in ice cold lysis buffer (1% Nondiet P-40, 25 mM Tris-HCl pH 7.2, 150 mM NaCl, 1 mM EDTA, 1 mM phenylmethylsulfonyl fluoride, 20 μg/ml aprotinin) 48 hours after the transfection. The cleared cell lysates were immunoprecipitated with anti-p100 antibody (C-5). The precipitated proteins or the total cell extracts were separated by SDS-polyacrylamide gel electrophoresis, transferred to a polyvinylidene difluoride membrane, and probed with an anti-Tax1 antibody (Taxy-7) [37], an anti-p100 antibody, or an anti-α-Tubulin antibody (DM1A), followed by visualization using the ECL Western blotting detection system (GE health science). The anti-p100 antibody (C-5) and the anti-α-Tubulin (DM1A) antibody were purchased from Santa Cruz Biotechnology and Calbiochem, respectively. NE-PER nuclear and cytoplasmic extraction reagents (Thermo Scientific) were used to prepare the cytoplasmic and nuclear lysates from Jurkat cells infected with the lentiviruses. The cytoplasmic (10 μg) and the nuclear (5 μg) lysates were characterized by a Western blotting analysis as described above.

### Transformation assay

The IL-2-independent transformation assay was conducted as previously described [38]. Briefly, CTLL-2 the cells were infected with lentiviruses encoding Tax1 or the indicated mutants and cultured in 96-well plates (1 × 10^3^, 3 × 10^3^, and 1 × 10^4^ cells/well) without IL-2 for four weeks. The number of wells containing outgrowing cells was counted using light microscopy.

## Competing interests

The authors declare that they have no competing interests.

## Authors' contributions

HT and MH designed the study, and performed the most of analysis. RK and MT produced Tax mutant constructs. YT provided the anti-Tax antibody. MF made substantial contributions to the conception and design of the study, wrote and drafted the manuscript, and contributed to data interpretation. MO and YA contributed to data interpretation. All authors read and approved the final manuscript.
